# Synergistic Effect of Nanoplastics and GenX on Human Serum Albumin: The Role of Protein Corona Formation and Co-Adsorption

**DOI:** 10.3390/toxics14010012

**Published:** 2025-12-22

**Authors:** Yuntao Qi, Qianyue Yin, Penghang Ni, Wansong Zong, Qigui Niu, Rutao Liu

**Affiliations:** 1 School of Environmental Science and Engineering, Shandong University, China-America CRC for Environment & Health, 72# Jimo Binhai Road, Qingdao 266237, China; 2 College of Geography and Environment, Shandong Normal University, 88# East Wenhua Road, Jinan 250014, China

**Keywords:** NPs, GenX, combined toxicity, molecular mechanism

## Abstract

GenX, also known as hexafluoroepoxypropane dimer acid (HFPO-DA), an emerging perfluoroalkyl substance alternative, is extensively used in industrial processes and is resistant to degradation. This persistence heightens the potential for co-occurrence and combined toxicity with other environmental pollutants. Nanoplastics (NPs), ubiquitous environmental contaminants, can exacerbate the biological toxicity of GenX. However, the molecular mechanisms by which NPs influence GenX-induced structural damage to human serum albumin (HSA) remain unclear. This study, therefore, employed multi-spectroscopic techniques, characterization assays, and molecular simulations to investigate these mechanisms. A critical limitation is that the observed structural damage occurred at a GenX concentration of 0.05–0.1 mM. The results indicate that the presence of NPs exacerbated the loosening of the protein backbone and caused a more pronounced reduction in *α*-helical content (NPs@GenX: 37.3%; GenX alone: 41.5%). The binding is predicted to occur within the hydrophobic pocket of subdomain IIIA of HSA. Characterization assays further revealed significant protein aggregation in systems containing NPs. The study concludes that NPs adsorb HSA through the formation of a protein corona, while simultaneously binding GenX via hydrophobic interactions. This dual pathway—direct binding of HSA to GenX and an active surface-mediated perturbation by NPs—constitutes the primary mechanism leading to aggravated structural changes. Overall, this work elucidates the molecular mechanisms by which NPs exacerbate HSA denaturation in the presence of GenX, offering valuable insights for assessing the combined ecological risks of emerging and persistent environmental pollutants.

## 1. Introduction

The widespread application of emerging contaminants poses severe environmental challenges. Plastics, serving as a cornerstone of modern industry due to their customizable physicochemical properties, exhibit an annual global production growth rate of 4% [1,2]. However, over half ultimately enter natural systems. Within this context, nanoplastics (NPs), formed as secondary environmental pollutants via photochemical degradation and mechanical fragmentation (diameter < 1 μm) [3], NPs can act as carriers in the environment, adsorbing hydrophobic organic pollutants (such as GenX) or heavy metals through their highly reactive interfaces to form composite pollutants (NPs@GenX) [4,5]. This complexation not only alters the environmental behavior and fate of individual pollutants but may also enhance their bioavailability [6]. For instance, the small-size effect and Brownian motion-dominated characteristics of NPs enable their stable suspension in water bodies, facilitating long-distance migration and increasing opportunities for contact with organisms [7,8,9]. Possess highly reactive interfaces capable of efficiently concentrating hydrophobic toxicants [10]. For example, Polystyrene nanoplastics amplify the toxic effects of PFOA on the Chinese mitten crab [11]. This raises concerns regarding potentially amplified toxicological effects.

GenX, a perfluorinated compound substitute for legacy PFOA, is seeing rapidly expanding use [12]. This shift is driven by the listing of perfluorooctane sulfonate (PFOS) and PFOA as persistent organic pollutants under the Stockholm Convention [13], prompting their phased elimination from numerous consumer and industrial processes [14]. GenX exhibits higher water solubility compared to PFOA; it is widely detected in water sources at varying levels around the world [15]. Even remote polar regions are not exempt, as HFPO-DA has been identified in Arctic surface water and Antarctic meltwater, albeit at significantly lower concentrations of 30 pg/L and 9.2 pg/L, respectively [16]. In China, HFPO-DA has been detected in Chinese groundwater (1.04–528 ng/L) [17]. Recent environmental studies indicate that GenX exhibits stronger adsorption stability onto microplastics (MPs) in lake water compared to PFOA [18]. Under environmentally relevant concentrations, the increased hybridization of MPs significantly enhances the bioaccumulation of both PFOA (k = 0.462) and GenX (k = 0.455) in E. crassipes [19,20]. This has raised concerns regarding their co-migration and the resulting combined exposure risks. Existing studies indicate that co-exposure to MPs and GenX induces marked intestinal tissue damage, characterized by elevated pro-inflammatory cytokine levels, altered antioxidant enzyme activities, and impaired expression of gut barrier proteins in the colon [21]. Nano-plastics possess a larger specific surface area than microplastics, enabling them to adsorb more pollutants [22]. Therefore, investigating the combined toxicity of NPs and GenX exposure is of greater importance. However, a critical knowledge gap persists regarding the underlying molecular mechanisms, particularly how NPs modulate conformational damage to essential functional proteins by GenX. Given the demonstrated capacity of NPs to carry pollutants into circulatory systems and form protein coronas [23], and that they can introduce contaminants into the circulatory system [24], interactions between NPs@GenX complexes and biological proteins appear inevitable. Consequently, assessing the NP-potentiated protein toxicity of GenX holds significant scientific value.

HSA, as the most abundant protein in human blood plasma with a concentration range of 35–50 g/L, serves as the principal carrier for numerous endogenous and exogenous substances, including the emerging contaminant GenX [25], Due to its structural sensitivity to a wide range of ligands and its well-characterized binding properties, HSA is widely established as a sentinel protein in mechanistic toxicology studies, making it a robust model for investigating initial molecular interactions and potential toxicity pathways. The binding affinity between HSA and GenX directly governs the in vivo distribution, bioavailability, and metabolic fate of GenX, thereby significantly influencing its potential toxicological outcomes [26]. Notably, environmental monitoring studies have revealed that GenX and NPs can coexist in adsorbed states in aquatic systems, leading to the formation of NPs@GenX composite pollutants. These composite entities can enter biological cycles through various pathways, raising concerns about their combined effects [25].

This study systematically investigates the synergistic amplification mechanism by which NPs exacerbate GenX-induced structural damage to HSA employing ultraviolet absorption, fluorescence spectroscopy, synchrotron radiation circular dichroism, and surface plasmon resonance techniques.

## 2. Materials and Methods

### 2.1. Chemicals

PS NPs (Polystyrene, 100 nm particle size) were obtained from Tianjin BaseLine Chromatography Technology Development Centre (Tianjin, China). GenX (CAS: 13252-13-6, purity ≥ 97%) was sourced from Aladdin Co., Ltd. (Shanghai, China). Human Serum Albumin (HSA) was procured from Solarbio Science & Technology Co., Ltd. (Beijing, China). Sodium phosphate buffer components (Na_2_HPO_4_·12H_2_O and NaH_2_PO_4_·2H_2_O) were purchased from Sinopharm Chemical Reagent Co., Ltd. (Shanghai, China). Ultrapure water (18.25 MΩ·cm) was supplied by a Millipore Milli-Q purification system.

### 2.2. HSA Structural Analysis

The NPs@GenX complex and GenX solution were incubated with HSA at 310 K for 30 min, then transferred into quartz cuvettes (10 mm path length) alongside matched control solutions (without HSA). Control solutions served as reference blanks. Cuvettes were positioned in the sample and reference holders of a UV-2600 spectrophotometer (Shimadzu, Kyoto, Japan). Measurements were performed with a 0.5 nm sampling interval and 2 nm slit width after zeroing with ultrapure water. Absorbance changes were recorded across 190–450 nm. For the detailed procedure, see Text S1.

### 2.3. Secondary Structure Characterization

HSA secondary structure alterations were quantified using a J-1500 circular dichroism (CD) spectrometer (JASCO, Tokyo, Japan). Solutions were loaded into 1 mm path length cuvettes and scanned from 190–260 nm at 200 nm/min. Three scans were averaged per sample after subtracting buffer background signals.

### 2.4. Fluorescence Spectroscopy Analysis

HSA fluorescence properties were analyzed using an F-4600 spectrofluorometer (Htachi, Tokyo, Japan) with the following parameters:

- Intrinsic Fluorescence: The excitation wavelength was 280 nm, the emission wavelength was 290–450 nm (PMT voltage: 650 V; scan speed: 1200 nm/min; interval: 0.2 nm).
- The fluorescence spectral parameters were set as follows: Both emission and synchronous fluorescence spectra of the experimental system were recorded on a fluorescence spectrophotometer. The instrument was uniformly set with the following parameters: photomultiplier tube (PMT) voltage at 700 V, data interval at 0.2 nm, and scan speed at 1200 nm/min. The specific spectral acquisition parameters were as follows:

Emission spectra: Excitation wavelength at 280 nm, with an emission wavelength scan range of 290–450 nm.

Synchronous fluorescence spectra: Δλ = 15, 60 nm (fixed emission wavelength at 310 nm), with an excitation wavelength scan range of 250–350 nm.

- The resonance light scattering (RLS) spectra of the experimental system were acquired using a fluorescence spectrophotometer. The key parameters were set as follows: photomultiplier tube (PMT) voltage at 600 V, and a data sampling interval of 0.2 nm. A complete scan was performed over the wavelength range of 200–600 nm to obtain the full RLS spectrum. This method of synchronous scanning with a zero wavelength difference is a standard technique for measuring resonance light scattering spectra using a conventional fluorescence spectrophotometer.
- 3D Fluorescence: λ_ex_ = 200–370 nm, λ_em_ = 250–600 nm (slit width: 5 nm; PMT: 550 V; data interval: 2 nm).

### 2.5. Hydrodynamic Size and Zeta Potential

Zeta potentials of GenX, NPs, and NPs@GenX systems were determined using a Zetasizer Nano-ZS90 (Malvern Panalytical Ltd., Malvern, UK). Size distributions were characterized via dynamic/static light scattering on a BI-200SM instrument (Brookhaven Instruments Corporation, Nashua, NH, USA). The raw data is presented in Figures S1–S3.

### 2.6. Molecular Docking Simulations

Molecular docking was performed with Molecular Operating Environment (MOE) 2014 Edition(Chemical Computing Group Inc., Montreal, QC, Canada) to investigate the binding modes of GenX complexes with HSA [27,28]. The HSA crystal structure (PDB: 1AO6) was acquired from the RCSB Protein Data Bank, and ligand structures were sourced from ChemicalBook. The protein was prepared by removing water molecules and adding hydrogen atoms. Potential binding sites were identified by the Site Finder tool, and interactions with amino acids were analyzed using MOE-Dock simulations.

### 2.7. Statistical Analysis

Data represent means ± SEM of three independent experiments. Statistical significance was assessed by one-way ANOVA with Tukey’s post hoc test using SPSS (v25.0, IBM Corp., New York, NY, USA).

## 3. Results and Discussion

### 3.1. Conformational and Structural Damage of HSA Induced by NPs@GenX

**Figure 1:**
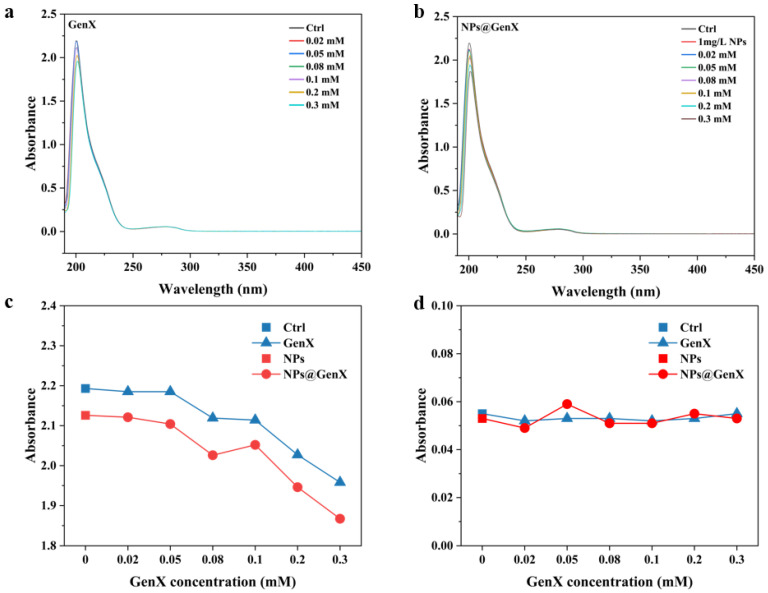
Changes in UV-Vis absorption spectra of HSA induced by (**a**) GenX, (**b**) NPs@GenX, (**c**) peak spectrum at 200 nm, and (**d**) peak spectrum at 280 nm.

Changes in the structure of biomacromolecules are routinely detected using ultraviolet-visible absorption spectroscopy [29]. In the HSA, the absorption peak at 200 nm is caused by the π–π * electron transition of C=O in the peptide chain, which is the backbone absorption peak of HSA [30]. The absorption peak around 280 nm indicates changes in the amino-acid microenvironment [31]. As illustrated in Figure 1, increasing concentrations of GenX exposure induced a concentration-dependent decrease in the absorbance peak at approximately 200 nm. This spectral shift indicates a conformational alteration in the HSA backbone, signifying structural loosening [32,33]. Exposure to NPs alone elicited a similar reduction in peak intensity at 200 nm. Notably, the decrease observed in the NPs@GenX combined exposure group was significantly more pronounced than that in the GenX-only exposure group. Under all exposure conditions, the absorbance peak at approximately 280 nm showed no significant shift, with both its position and intensity remaining near the control level [31]. This stability suggests that the microenvironment surrounding the aromatic amino acid residues of HSA was unaffected by either GenX, NPs, or their combination [34]. Collectively, these results demonstrate that NPs exacerbate the structural perturbation of HSA induced by GenX, ultimately leading to a more substantial degree of protein structural loosening.

To further explore the structural changes of HSA, we tested its protein secondary structure [35]. As the basis of protein structure, the results are shown in Table 1. In the control group, *α*-helices in HSA account for 44.8%, *β*-sheets for 20%, *β*-turns for 7.1%, and random coils for 27.3%. After exposure to GenX, the proportion of *α*-helices decreased to 43.8 and 41.5, respectively, while the proportion of *β*-sheets increased to 18.7% and 19.9%. After exposure to NPs, the proportion of *α*-helices decreased to 42.1%. However, in the NPs@GenX system, the proportion of *α*-helices decreased to 38.4% and 37.3%, respectively, and the proportion of random coils increased, indicating that the introduction of NPs may cause the disorder of the peptide chain [36]. Alterations in the secondary structure of HSA are likely to compromise its tertiary conformation and functional integrity [36,37]. The significantly greater reduction in the 200 nm peak absorbance observed in the NPs@GenX complex group (Figure 1), compared to either GenX or NPs alone, indicates a more pronounced disruption of the protein’s backbone structure. This enhanced perturbation likely arises from synergistic interactions between NPs and GenX. The nanoparticles may provide a high surface area for the adsorption and concentration of GenX molecules near the protein, facilitating their access to and interaction with critical regions of the HSA backbone. Consequently, exposure to the NPs@GenX complex induces more substantial structural destabilization in HSA than exposure to the individual components.

### 3.2. Effect of NPs@GenX on the Intrinsic Fluorescence of HSA

**Figure 2:**
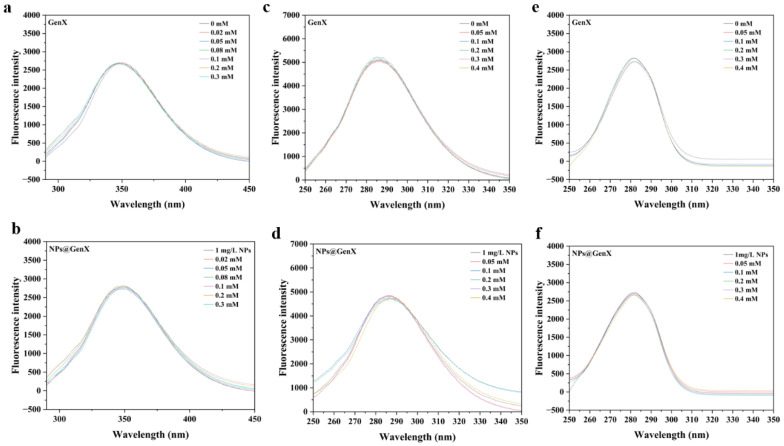
Displays the (**a**,**b**) endogenous, (**c**,**d**) Tyr (Δλ = 60 nm), and (**e**,**f**) Trp (Δλ = 15 nm) fluorescence spectra of HSA after exposure to NPs, NPs@GenX, and GenX, under the conditions of C_HSA_ = 10^−6^ M, T = 310 K, and pH = 7.4.

Fluorescence spectroscopy is used to investigate the changes in fluorescent amino acid residues [38], which in turn reflect alterations in the overall protein structure. In HSA, the intrinsic fluorescence primarily originates from the tryptophan (Trp) residue [39]. Prior to the formal experiments, a preliminary test was conducted in Figure 2, which revealed that the difference in absorption values of GenX at the excitation and emission peak wavelengths was less than 5% [40]. Therefore, the inner filter effect could be considered negligible. As shown in Figure 2a,b, neither NPs nor GenX alone induced significant changes in the fluorescence intensity, and their combination also showed no notable effect, indicating that neither GenX nor NPs substantially influenced the chromophore environment of HSA.

To further investigate the fluorescence status of specific amino acid residues, synchronous fluorescence spectroscopy was also performed [41]. The results were consistent with those of the conventional fluorescence measurements [42], confirming that exposure to GenX and NPs, either individually or in combination, did not induce significant conformational changes in HSA. These findings are in good agreement with the UV absorption results, which showed no obvious alteration in the absorption peak at 280 nm, further supporting the conclusion that the structure of HSA remains largely unaffected under the experimental conditions.

### 3.3. Effect of NPs@GenX on the Three-Dimensional Fluorescence Spectra of HSA

Three-dimensional fluorescence spectroscopy was employed to investigate the conformational changes of HSA under various exposure conditions. The 3D fluorescence spectra of HSA exhibit two characteristic peaks: Peak 1 primarily reflects the microenvironmental alterations of aromatic amino acid residues such as tryptophan (Trp) and tyrosine (Tyr), while Peak 2 represents the structural features of the polypeptide backbone [43].

**Figure 3:**
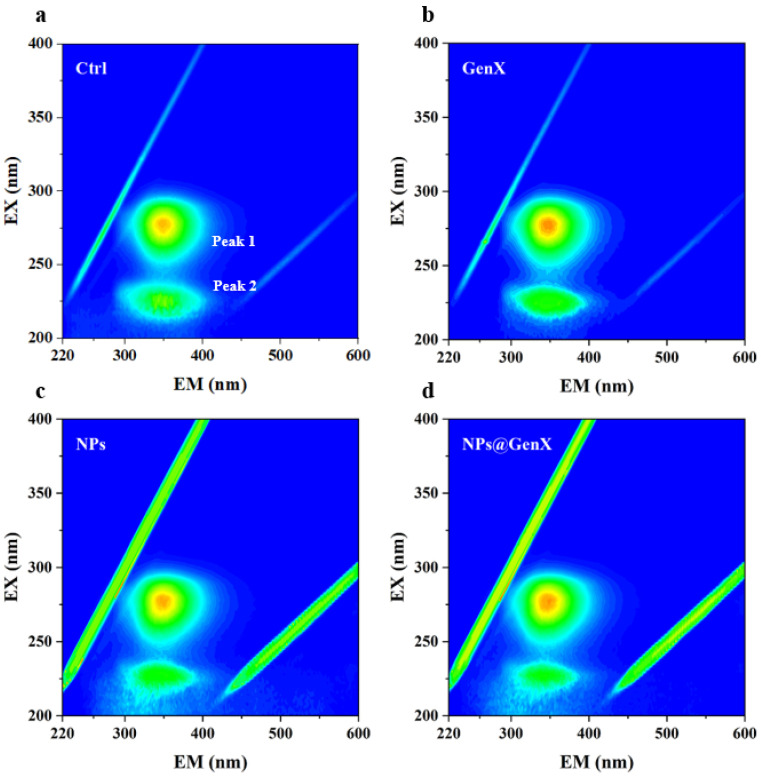
3D fluorescence spectroscopy of HSA induced by (**a**) control, (**b**) GenX, (**c**) NPs, and (**d**) NPs@GenX. C_HSA_ = 10^−6^ M.

As shown in Figure 3, Three-dimensional fluorescence spectra demonstrated: Peak 1 showed no significant alterations across GenX, NPs, and NPs@GenX groups, confirming stable Trp/Tyr microenvironments. Peak 2 exhibited reduced intensity with broadening exclusively under NPs@GenX exposure, indicating backbone conformational loosening. The Rayleigh scattering peak displayed markedly elevated intensity in the NPs group but attenuated enhancement in NPs@GenX. Collectively, NPs and GenX act synergistically to disrupt HSA secondary structure and alter aggregation patterns, exacerbating protein backbone destabilization.

### 3.4. Analysis of Particle Size and Stability in the NPs@GenX-HSA System

To further investigate the specific binding mechanism between NPs@GenX and HSA, DLS, RLS, and Zeta potential measurements were employed. These techniques were used to evaluate the aggregation state, hydrodynamic diameter, and binding stability of the system, respectively [44,45]. Together, these methods provide a comprehensive analysis of the “charge–conformation–aggregation” relationship.

**Figure 4:**
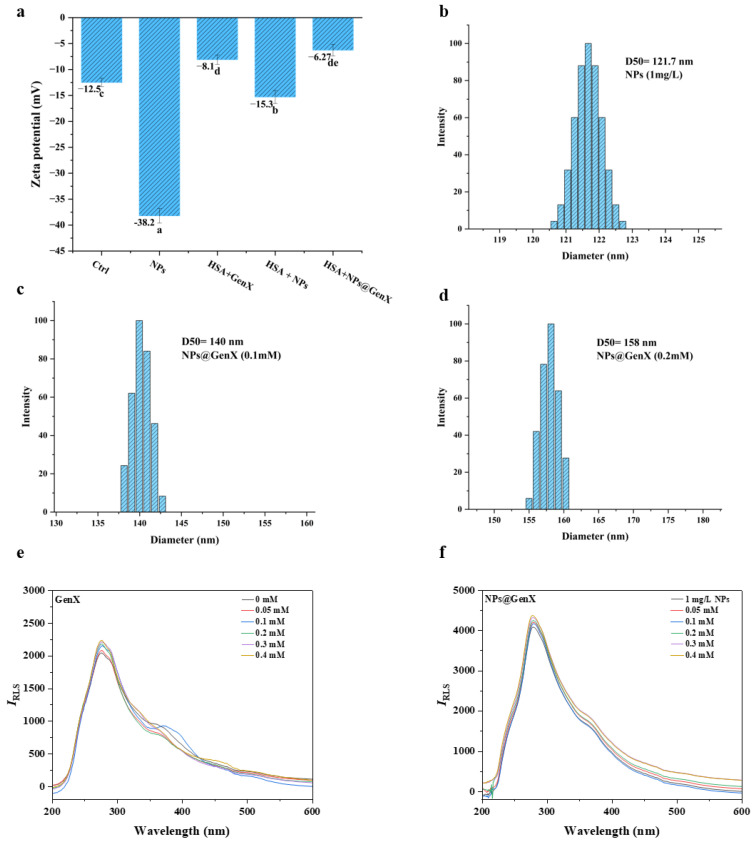
(**a**) The Zeta potential of each mixed system, C_(GenX)_: 0.1 mM C_(NPs)_: 1 mg/L, Different lowercase letters indicate significant differences exist; (**b**–**d**) the DLS changes in the NPs@GenX system; (**e**,**f**) the RLS changes in the GenX and NPs@GenX systems. C_HSA_ = 10^−6^ M.

The Zeta potential results are shown in Figure 4. Both HSA and NPs alone exhibited negative charges, with Zeta potentials of −12.5 mV and −38.2 mV, respectively. However, after binding, the absolute value of the Zeta potential decreased significantly to −6.27 mV in the NPs@GenX group, indicating a substantial reduction in system stability.

Dynamic light scattering results indicate that particle size in the NPs@GenX group increases with rising GenX concentration, suggesting the formation of larger aggregates due to interactions between NPs@GenX particles. To further substantiate this, we tested the DLS of a mixed system comprising NPs and HSA, as shown in Figure S4. The agglomeration of particle size in this system further indicates the formation of a protein crown.

Meanwhile, RLS experiments demonstrated that both GenX alone and NPs@GenX induced particle aggregation. The RLS intensity of the NPs group increased significantly, indicating that the high surface free energy of NPs promotes their binding with proteins. The increase in particle size, as reflected by RLS, suggests the formation of protein-particle complexes through the attachment of proteins to NPs@GenX particles.

In summary, the combination of DLS, RLS, and Zeta potential measurements consistently demonstrates that NPs@GenX interacts with HSA, leading to reduced stability, increased particle size, and aggregation, thereby elucidating the binding mechanism and its impact on the protein’s behavior in solution.

### 3.5. Simulation of GenX Binding Sites on HSA

Molecular docking serves as a powerful computational method for elucidating interaction mechanisms between proteins and small molecules [46]. Previous experimental results from various techniques indicated that while GenX binding to HSA did not induce significant fluorescence changes, it caused noticeable alterations in the protein’s secondary structure. This evidence suggests that GenX likely binds to Sudlow site II of HSA. It is important to note that the proposed interaction mechanism is derived from studies using isolated HSA, and its extrapolation to more complex cellular or in vivo systems requires further validation.

**Figure 5:**
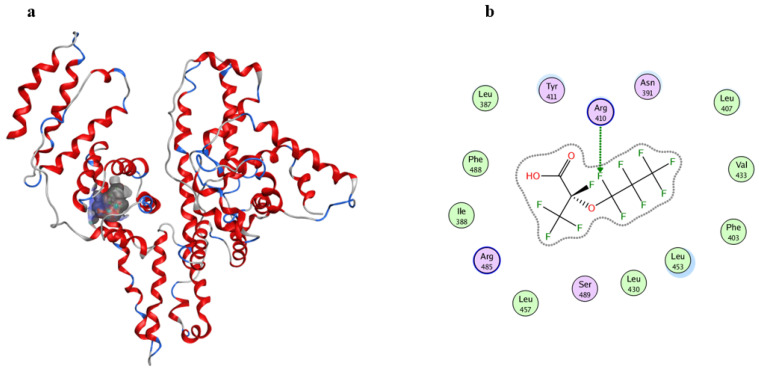
(**a**) Predicted GenX-HSA binding model; (**b**) Gaussian contact pattern map showing ligand–receptor superposition and hydrogen bond. The letters in (**b**) represent amino acids, and the arrows indicate the amino acids primarily involved in binding between HSA and GenX.

To further characterize the binding site and its molecular environment, we performed molecular docking simulations. As shown in Figure 5, results demonstrate that the perfluoroalkyl chain of GenX inserts into the hydrophobic cavity of subdomain IIIA, formed by residues including Leu387, Leu453, and Phe403. Additionally, GenX forms a hydrogen bond with Arg410 (bond length: 2.7 Å (Figure S5)). These interactions provide a structural explanation for the observed conformational changes in HSA.

Notably, NPs are substantially larger than HSA and therefore cannot directly access its internal binding sites [20,47]. However, based on previous experimental evidence, we propose that the supra-environmental concentration of GenX used in this mechanistic study, once adsorbed on NP surfaces, remains available for interaction with HSA. This configuration facilitates direct binding of GenX to the protein, potentially leading to enhanced structural perturbation. In this process, HSA forms a protein corona on the NPs@GenX complex [48]. Thus, the structural integrity of HSA is affected through a dual mechanism: direct interaction with GenX and indirect effects mediated by nanoparticle surfaces. These findings elucidate a potential molecular mechanism, without direct inference to environmental or human health risks.

## 4. Conclusions

This study clarifies that the NPs@GenX complex causes more pronounced structural damage to HSA than its individual components, evidenced by a greater reduction in α-helical content (37.3% vs. 41.5% for GenX alone) and an increase in random coil structures. DLS, RLS, and Zeta potential measurements confirmed that this complex promotes the formation of larger HSA aggregates and reduces protein stability. Based on the observed synergistic effects and the known hydrophobic adsorption between NPs and GenX, we propose a potential dual-interaction scenario (simultaneous solution binding and NP-surface mediation) that may collectively amplify structural damage.

## Figures and Tables

**Table 1:** HSA secondary structure changes by GenX, NPs, and NPs@GenX. C_HSA_ = 10^−6^ M, T = 310 K, and pH = 7.4.

Treatments	Concentration	Secondary Structural Contents in HSA (%)			
		*α*-Helix	*β*-Sheet	Turn	Random
Control	0 mM	44.8	17.7	9.1	26.3
NPs	1 mg/L	42.1	28.2	3.9	25.8
GenX	0.05 mM	43.8	18.7	9.2	28.3
	0.1 mM	41.5	19.9	8.5	25
NPs@GenX	0.05 mM	38.4	19.6	8.9	33.1
	0.1 mM	37.3	20.1	11.7	30.9

## Data Availability

Data is contained within the article or Supplementary Materials.

## Supplementary Materials

The following supporting information can be downloaded at: https://www.mdpi.com/article/10.3390/toxics14010012/s1, Text S1: The Process of NPs and GenX Complex Formation; Figure S1: NPs, DLS Measurement Data and Functions; Figure S2: NPs@GenX (0.1 mM), DLS Measurement Data and Functions; Figure S3: NPs@GenX (0.2 mM), DLS Measurement Data and Functions; Figure S4: DLS of Nanoplastics Combined with HAS; Figure S5: Binding Energy in Molecular Docking [49].
