# Supplementary material for: Synergistic Effect of Nanoplastics and GenX on Human Serum Albumin: The Role of Protein Corona Formation and Co-Adsorption
Source: Toxics. 2025 Dec 22;14(1):12. doi: 10.3390/toxics14010012 (PMC12845872; doi:10.3390/toxics14010012)
Supplement: Supplementary file 1 [file toxics-14-00012-s001.zip › toxics-4023508-supplementary.pdf]

Synergistic Effect of Nanoplastics and GenX on Human Serum Albumin: The Role of Protein Corona Formation and Co-Adsorption

Yuntao Qi [1], Qianyue Yin [1], Penghang Ni [1], Wansong Zong [2], Qigui Niu [1,*] and Rutao Liu [1,*]

[1] School of Environmental Science and Engineering, Shandong University, China-America CRC for Environment & Health, Shandong Province, 72# Jimo Binhai Road, Qingdao 266237, China

[2] College of Geography and Environment, Shandong Normal University, 88# East Wenhua Road, Jinan 250014, China

* Correspondence: niuqg@sdu.edu.cn (Q.N.); rutaoliu@sdu.edu.cn (R.L.); Tel./Fax: +86-0531-88365489 (R.L.)

Text S1. The Process of NPs and GenX Complex Formation

First, stock solutions of GenX at various concentration gradients and nanoplastics (NPs) at a fixed concentration (10 mg/L) were prepared using ultrapure water according to the experimental design. The NPs and GenX solutions were then mixed and incubated with gentle shaking (e.g., at 150 rpm) at room temperature for 48 hours to form stable composite pollutants (NPs@GenX). This pre-incubation step, confirmed by prior studies to facilitate reaching a stable interaction state [49], was employed before co-exposure with HSA. The main protein incubation system consisted of phosphate-buffered saline 0.02 mM, pH 7.4, GenX, human serum albumin (HSA), and ultrapure water. After mixing all components thoroughly to ensure complete dissolution, the reaction mixtures were incubated at 310 K (approximately 37°C) for 30 minutes before subsequent spectroscopic measurements.

Figure S1 NPs

DLS Measurement Data and Functions

Sample ID	0
Operator ID	Unknown Operator
Elapsed Time	00:00:31
Mean Diam.	121.7 (nm)
Rel. Var.	0.000
Skew	0.009
RmsError	1.6799e-03

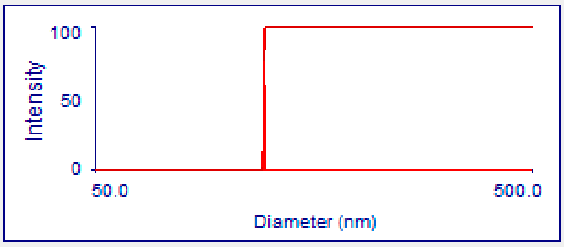

d	G(d)	C(d)	d	G(d)	C(d)	d	G(d)	C(d)
118.18	0	0	120.64	4	1	122.92	0	100
118.41	0	0	120.84	13	3	123.45	0	100
118.63	0	0	121.05	32	10	123.69	0	100
118.86	0	0	121.26	60	22	123.92	0	100
119.08	0	0	121.46	88	40	124.16	0	100
119.31	0	0	121.67	100	60	124.39	0	100
119.54	0	0	121.88	88	78	124.63	0	100
119.76	0	0	122.09	60	90	124.87	0	100
119.99	0	0	122.30	32	97	125.10	0	100
120.22	0	0	122.50	13	99	125.34	0	100
120.43	0	0	122.71	4	100	125.58	0	100

Print Window

Copy For Spreadsheet

Copy to Clipboard

Close

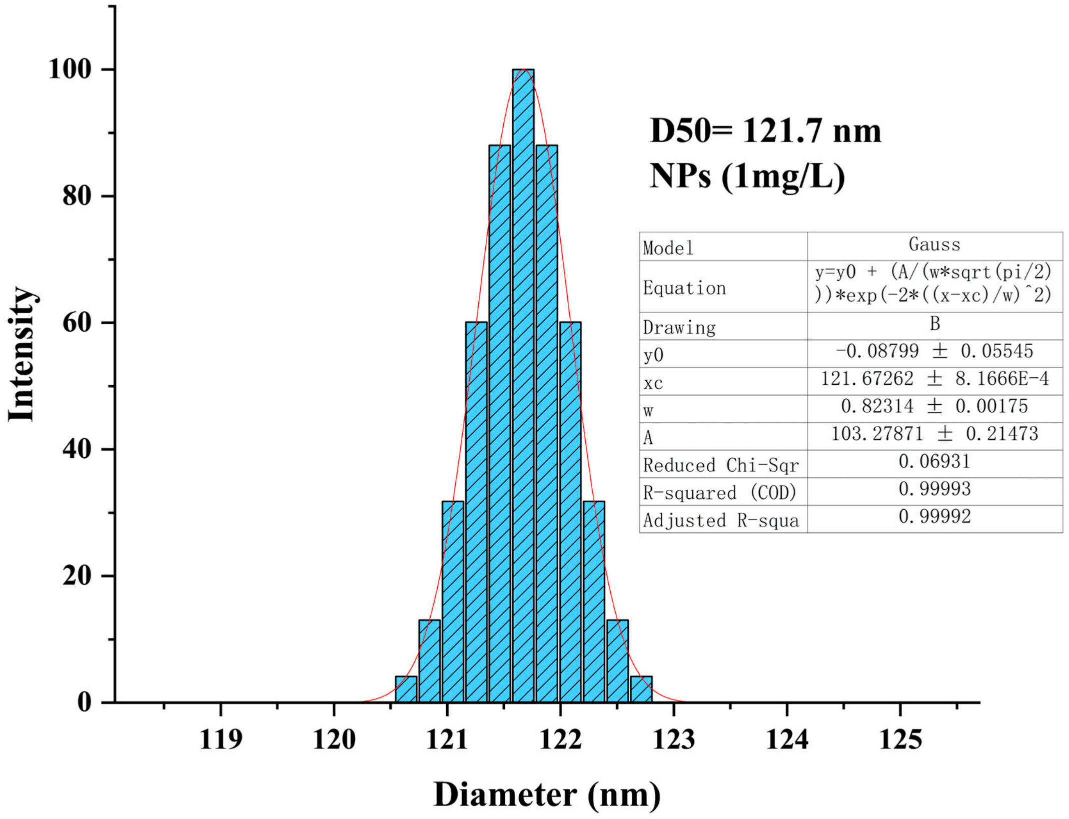

Model	Gauss
Equation	y=y0 + (A/(w*sqrt(pi/2)))*exp(-2*((x-xc)/w)^2)
Drawing	B
y0	-0.08799 ± 0.05545
xc	121.67262 ± 8.1666E-4
w	0.82314 ± 0.00175
A	103.27871 ± 0.21473
Reduced Chi-Sqr	0.06931
R-squared (COD)	0.99993
Adjusted R-squa	0.99992

Figure S2 NPs@GenX（0.1mM)

DLS Measurement Data and Functions

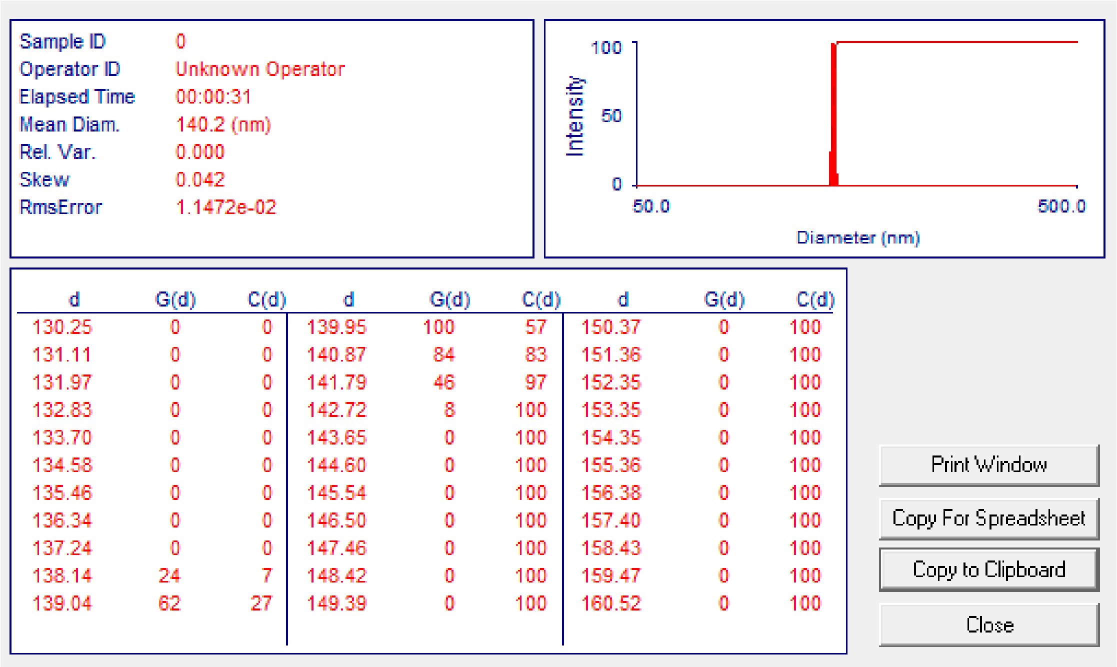

Sample ID	0
Operator ID	Unknown Operator
Elapsed Time	00:00:31
Mean Diam.	140.2 (nm)
Rel. Var.	0.000
Skew	0.042
RmsError	1.1472e-02

d	G(d)	C(d)	d	G(d)	C(d)	d	G(d)	C(d)
130.25	0	0	139.95	100	57	150.37	0	100
131.11	0	0	140.87	84	83	151.36	0	100
131.97	0	0	141.79	46	97	152.35	0	100
132.83	0	0	142.72	8	100	153.35	0	100
133.70	0	0	143.65	0	100	154.35	0	100
134.58	0	0	144.60	0	100	155.36	0	100
135.46	0	0	145.54	0	100	156.38	0	100
136.34	0	0	146.50	0	100	157.40	0	100
137.24	0	0	147.46	0	100	158.43	0	100
138.14	24	7	148.42	0	100	159.47	0	100
139.04	62	27	149.39	0	100	160.52	0	100

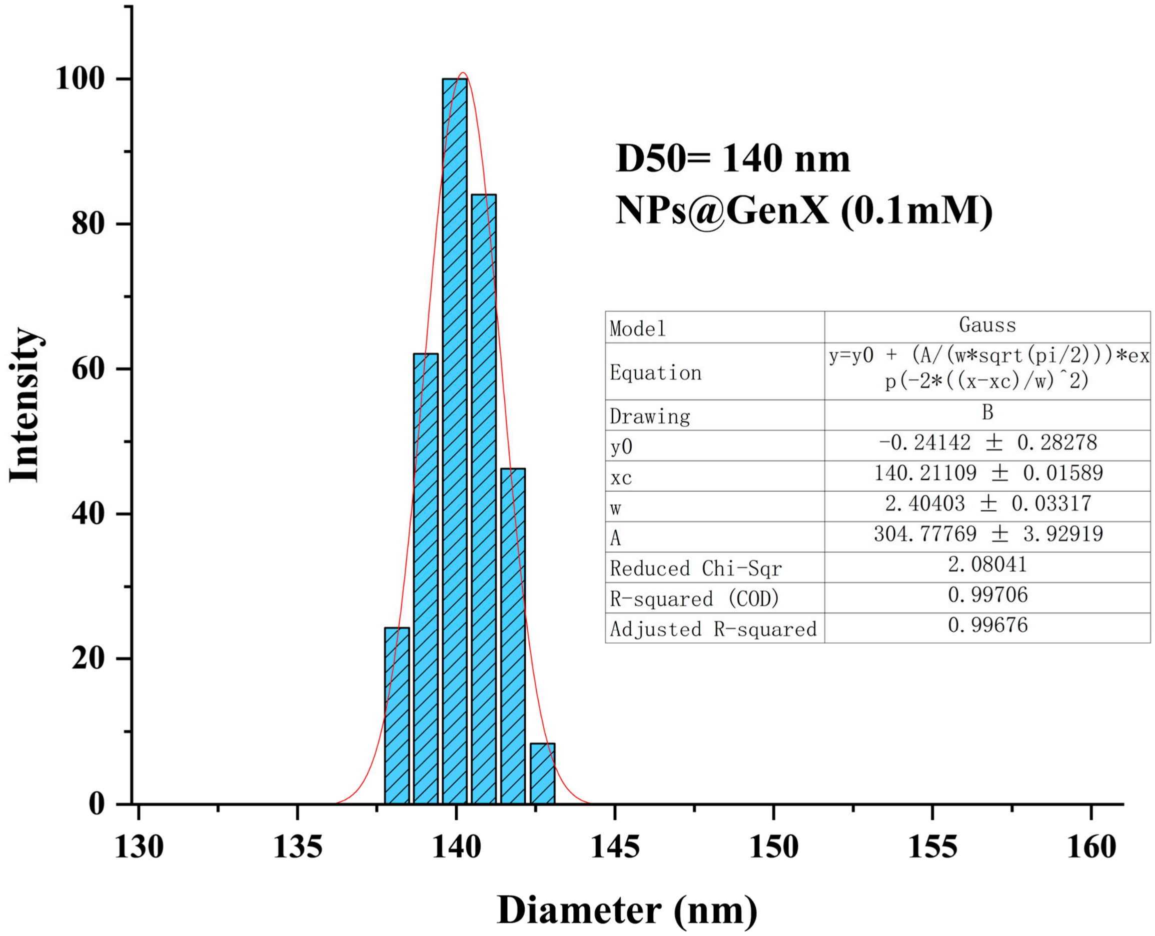

Model	Gauss
Equation	y=y0 + (A/(w*sqrt(pi/2)))*exp(-2*((x-xc)/w)^2)
Drawing	B
y0	-0.24142 ± 0.28278
xc	140.21109 ± 0.01589
w	2.40403 ± 0.03317
A	304.77769 ± 3.92919
Reduced Chi-Sqr	2.08041
R-squared (COD)	0.99706
Adjusted R-squared	0.99676

Figure S3 NPs@GenX（0.2mM）

DLS Measurement Data and Functions

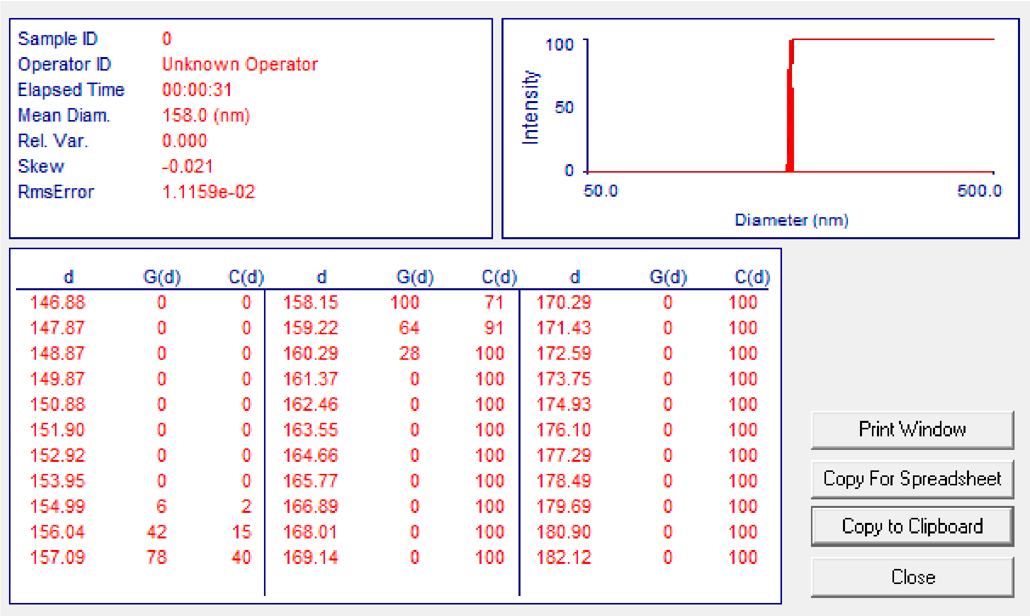

Sample ID	0
Operator ID	Unknown Operator
Elapsed Time	00:00:31
Mean Diam.	158.0 (nm)
Rel. Var.	0.000
Skew	-0.021
RmsError	1.1159e-02

d	G(d)	C(d)	d	G(d)	C(d)	d	G(d)	C(d)
146.88	0	0	158.15	100	71	170.29	0	100
147.87	0	0	159.22	64	91	171.43	0	100
148.87	0	0	160.29	28	100	172.59	0	100
149.87	0	0	161.37	0	100	173.75	0	100
150.88	0	0	162.46	0	100	174.93	0	100
151.90	0	0	163.55	0	100	176.10	0	100
152.92	0	0	164.66	0	100	177.29	0	100
153.95	0	0	165.77	0	100	178.49	0	100
154.99	6	2	166.89	0	100	179.69	0	100
156.04	42	15	168.01	0	100	180.90	0	100
157.09	78	40	169.14	0	100	182.12	0	100

Print Window
Copy For Spreadsheet
Copy to Clipboard
Close

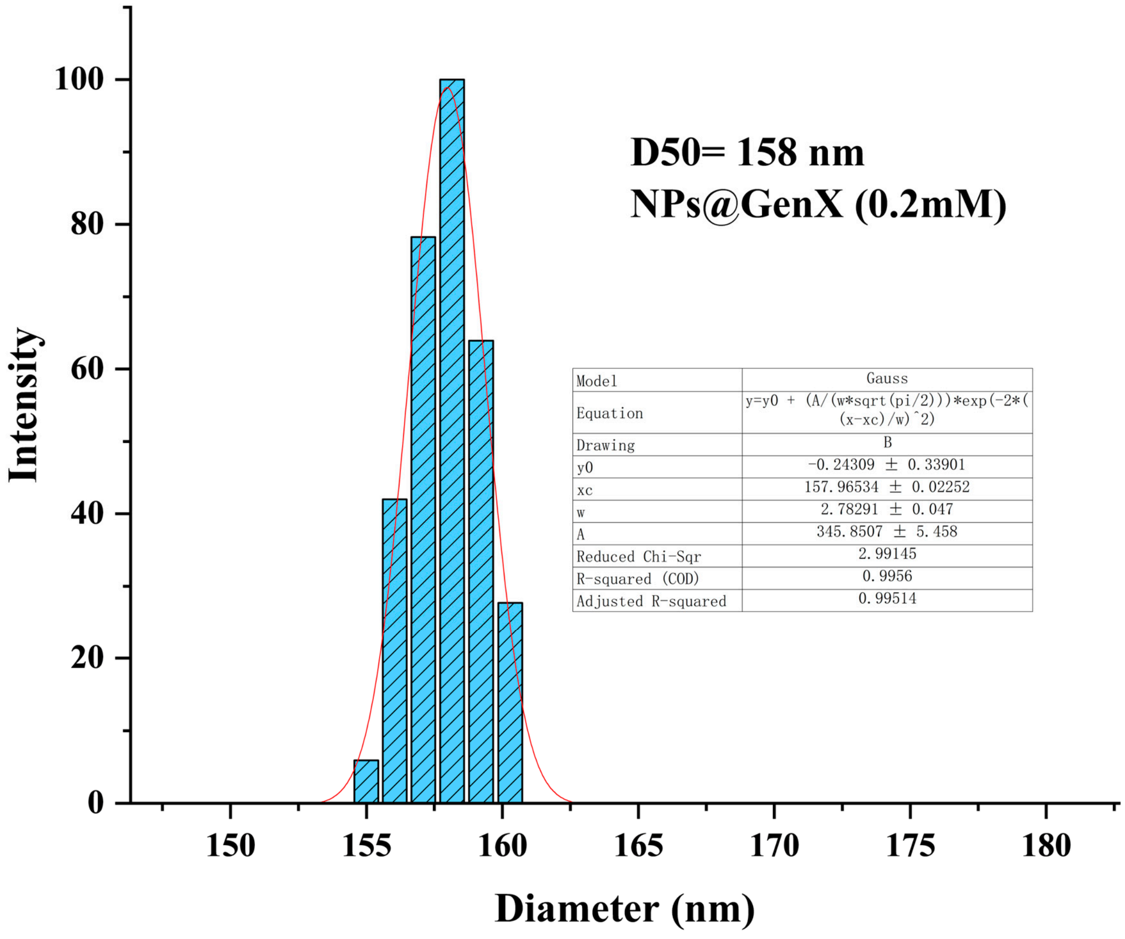

Model	Gauss
Equation	y=y0 + (A/(w*sqrt(pi/2)))*exp(-2*((x-xc)/w)^2)
Drawing	B
y0	-0.24309 ± 0.33901
xc	157.96534 ± 0.02252
w	2.78291 ± 0.047
A	345.8507 ± 5.458
Reduced Chi-Sqr	2.99145
R-squared (COD)	0.9956
Adjusted R-squared	0.99514

Figure S4 DLS of Nanoplastics Combined with HSA

NPs+HSA

Sample ID	0
Operator ID	Unknown Operator
Elapsed Time	00:01:00
Mean Diam.	208.6 (nm)
Rel. Var.	0.035
Skew	-2.726
RmsError	5.0241e-04

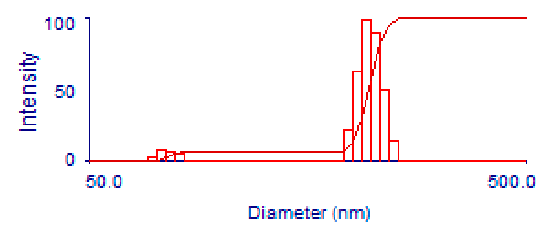

d	G(d)	C(d)	d	G(d)	C(d)	d	G(d)	C(d)
60.39	0	0	103.22	0	7	176.42	0	7
63.41	0	0	108.37	0	7	185.23	0	7
66.57	0	0	113.78	0	7	194.47	23	13
69.90	4	1	119.46	0	7	204.19	65	31
73.39	9	3	125.43	0	7	214.38	100	58
77.05	8	5	131.69	0	7	225.09	92	82
80.90	6	7	138.27	0	7	236.33	51	96
84.94	0	7	145.17	0	7	248.13	15	100
89.18	0	7	152.42	0	7	260.52	0	100
93.63	0	7	160.03	0	7	273.53	0	100
98.31	0	7	168.03	0	7	287.18	0	100

Print Window
Copy For Spreadsheet
Copy to Clipboard
Close

Figure S5 Binding Energy in Molecular Docking

```
XXXX: CARRIER PROTEIN / XXXX

Ligand       Receptor              Interaction  Distance  E (kcal/mol)
F     16     NH2     ARG  410  (A)  H-acceptor     2.70       -1.0
```

CD master data

The CD contains large amounts of raw data. If needed, please contact us via email. Here we provide images of the CD opened and analyzed using CD Pro.

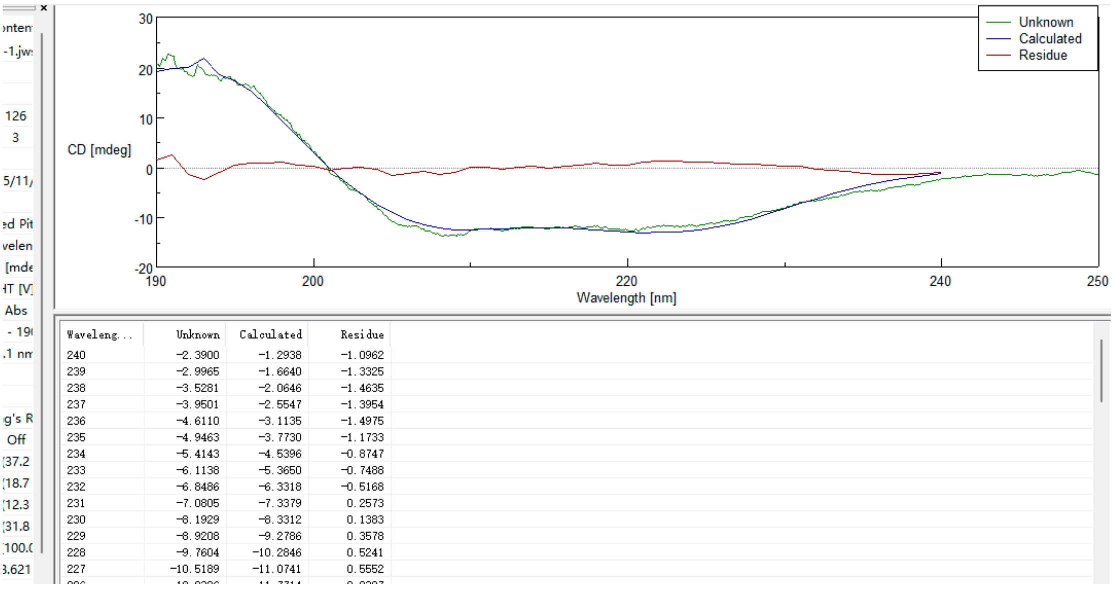

Waveleng...	Unknown	Calculated	Residue
240	−2.3900	−1.2938	−1.0962
239	−2.9965	−1.6640	−1.3325
238	−3.5281	−2.0646	−1.4635
237	−3.9501	−2.5547	−1.3954
236	−4.6110	−3.1135	−1.4975
235	−4.9463	−3.7730	−1.1733
234	−5.4143	−4.5396	−0.8747
233	−6.1138	−5.3650	−0.7488
232	−6.8486	−6.3318	−0.5168
231	−7.0805	−7.3379	0.2573
230	−8.1929	−8.3312	0.1383
229	−8.9208	−9.2786	0.3578
228	−9.7604	−10.2846	0.5241
227	−10.5189	−11.0741	0.5552

[49] B. Barhoumi, S.G. Sander, I. Tolosa, A review on per- and polyfluorinated alkyl substances (PFASs) in microplastic and food-contact materials, Environmental Research, 206 (2022) 112595.
